# Changes in volume and incidence of lymphedema during and after treatment with docetaxel, doxorubicin, and cyclophosphamide (TAC) in patients with breast cancer

**DOI:** 10.1007/s00520-017-3907-1

**Published:** 2017-11-10

**Authors:** Janine T. Hidding, Carien H. G. Beurskens, Philip J. van der Wees, Wilmy C. A. M. Bos, Maria W. G. Nijhuis-van der Sanden, Hanneke W. M. van Laarhoven

**Affiliations:** 1 Department of Orthopedics, Section of Physical Therapy, Radboud University Medical Center, Nijmegen, The Netherlands; 2 Radboud Institute for Health Sciences, Scientific Institute for Quality of Healthcare (IQ Healthcare), Radboud University Medical Center, Geert Grooteplein Noord 21, 6525 EZ Nijmegen, The Netherlands; 3 Department of Medical Oncology, Radboud University Medical Center, Nijmegen, The Netherlands; 4 Academic Medical Center, Department of Medical Oncology, University of Amsterdam, Amsterdam, The Netherlands

**Keywords:** Breast neoplasms, Lymphedema, Adjuvant chemotherapy, Activities of daily living, Quality of life

## Abstract

**Purposes:**

The purposes of this study were to investigate the incidence of lymphedema in patients with breast cancer during and after adjuvant treatment with docetaxel, doxorubicin, and cyclophosphamide (TAC), to identify predictors for development of lymphedema, and to describe consequences in daily life in relation to lymphedema.

**Methods:**

This is a prospective study with measurements before chemotherapy (T0), during chemotherapy before cycle 2 (T1), cycle 4 (T2), and 1 month after completion of treatment (T3). Volume change was monitored using tape measurements. Lymphedema was defined as ≥ 10% volume difference. Linear mixed-effect models were estimated to analyze differences in arm volume and consequences in daily life (total score and domain scores of the Lymph-International Classification of Functioning (ICF) questionnaire) over time and to identify treatment and patient characteristics as predictors for changes in volume.

**Results:**

Forty-eight patients completed all measurements. Volume did not change during TAC treatment. One month after treatment, volume was significantly increased compared to T0-T2, and 12 patients (25%) had developed lymphedema. Axillary lymph node dissection was associated with lymphedema (ES 2.9, 95% CI 0.02–5.7; *p* < 0.05). In patients with and without lymphedema, 1 month after completion (T3), the Lymph-ICF questionnaire showed significant limitations in physical function compared to T0-T2. In patients with lymphedema at T3, a significant association between volume and total score on the Lymph-ICF questionnaire on physical function and mobility activities was observed.

**Conclusions:**

One month after treatment in 12 patients (25%), volume difference increased over 10%. Axillary lymph node dissection was predictive for development of lymphedema. All patients, but more patients with lymphedema, perceived difficulties in activities in daily life after treatment.

## Introduction

Lymphedema is a common side effect of breast cancer treatment, usually starting within 2 years after treatment [1]. Patients with lymphedema suffer not only from swelling but also from other impairments in functions and limitations in activities in daily life, as described in the core set lymphedema based on the International Classification of Functioning (ICF) [2]. Lymphedema is defined as a volume difference between upper extremities of ≥ 10% [3], resulting in limitations in arm use during daily activities, emotional distress, restrictions in social activities, and limited work abilities [2, 4, 5].

The estimated incidence of lymphedema 5 years after breast cancer treatment is 16.6%, and increase in arm volume is related to axillary lymph node dissection, the number of lymph nodes dissected, mastectomy, radiotherapy to the axilla, and a body mass index over 25 kg/m^2^ [1, 6]. Recent studies have indicated that the use of adjuvant cytotoxic treatment may be associated with development of lymphedema after completion of treatment, especially regimens containing anthracyclines and taxanes [6–14]. Swelling may decline over time, as a result of lymphedema treatment or due to spontaneous recovery of transient swelling within 3 months [13, 14]. Adjuvant chemotherapy has been shown to improve survival in patients with early-stage breast cancer. Second and third generation schemes are more effective in survival compared to first generation schemes [15]. A frequently used third generation regimen consists of docetaxel, doxorubicin, and cyclophosphamide (TAC) [16].

Although the prevalence of lymphedema in the arm after completion of TAC has been reported [11–14], development of lymphedema during treatment with TAC and limitations in daily activities in relation to lymphedema during and after TAC are unknown. Early detection of lymphedema and consequently early intervention can lessen treatment burden and increase the cost-effectiveness of care [17]. Therefore, it is clinically relevant to obtain insight in changes in volume differences or the amount of extracellular fluid in an early stage.

The purpose of this study is to answer the following questions: (1) what is the change in arm volume during adjuvant treatment with TAC, and do patients develop lymphedema as defined by a volume difference between upper extremities of ≥ 10%, (2) which predictors for development of lymphedema can be identified, and (3) which consequences in daily life are related to the presence of lymphedema?

## Methods

### Study design

We conducted a prospective cohort study in which patients with unilateral breast cancer were scheduled for adjuvant cytotoxic treatment with six cycles of TAC. Patients were measured at four time points: at baseline before cycle 1 (T0), during chemotherapy before cycle 2 (T1) and cycle 4 (T2), and 1 month after the 6th cycle (T3). Three months after completion, the Lymph-ICF questionnaire was sent to the patients (T4).

### Patient population

Patients, both female and male, with tumor stages I–III, scheduled for adjuvant cytotoxic treatment with TAC at the Radboud University Medical Center were invited by a specialized nurse (WB) to participate in this study. Patients were included between August 2011 and January 2015. Surgery was completed, as well as radiotherapy if indicated, before the start of TAC. Exclusion criteria were recurrence or second cancer and insufficient understanding of Dutch language for filling out the questionnaire. Formal ethical approval was waived by the Medical Ethical Committee of the Radboud University Medical Center. The study was registered under number 2011/234. All participants signed informed consent before the first measurement. We calculated the sample size based on arm volume difference as primary endpoint. A priori, a dropout of 10% was taken into account. To detect changes at a two-sided significance level of 5% and an estimated power of 80%, we planned to enroll 50 patients.

### Chemotherapy

Docetaxel (75 mg/m^2^), doxorubicin (50 mg/m^2^), and cyclophosphamide (500 mg/m^2^) were administered intravenously on day 1 of a three-weekly cycle for a total of six courses. Dexamethasone was administered 8 mg orally twice daily for 3 days, starting the day before start of TAC during all cycles.

### Measurements

Demographic and tumor characteristics, type of surgery, axillary lymph node dissection, tumor stage, nodal stage, tumor grade, adjuvant radiotherapy, radiotherapy to the axilla or supraclavicular region, weight, and height were derived from electronic health records of the included patients. Early termination of TAC or dose reduction was registered, as well as the reason for the early stop and change of cytotoxic agents. Weight was registered before the first and after the last cycle to determine body mass index (BMI) and weight changes.

Volume of both arms was measured by tape measurement [18], and impairments in functions and limitations in activities in daily life were measured by the Lymph-ICF questionnaire [19]. Measurements were performed by three physiotherapists (CB, RD, JH), experienced in measuring arm volume. Investigators were blinded for results of prior measurements. The measurement protocol was described in detail to reduce measurement error and, if possible, patients were measured by the same therapist throughout the whole study. All patients with a volume difference ≥ 10%, at any time point, were referred for treatment. Both upper limbs were measured by tape measurement with 10 cm intervals up to 40 cm, starting at the ulnar styloid process. Position of the arm during measurement was in 90° flexion of the shoulder with the elbow extended. Hands were supported on a pillow. To calculate volume, the conical formula was used [18]. Volumes between both upper extremities were converted to percentage differences (relative volume, in short mentioned as volume in this article). Tape measurement is a reliable measurement instrument, with excellent intra- and intertest-retest reliability (ICC 0.99 and 0.98, respectively) and good validity (0.80–1.00) compared to water volumetry when a standardized protocol is used [18].

Volume differences, based on the tape measurement, were computed at all four time points. A volume difference ≥ 10% between both upper extremities was indicated as swelling or development of lymphedema [3]. To get insight in outcomes between patients without and with lymphedema 1 month after completion, and to get insight in the association between volume differences and impairments in functions and limitations in activities in daily life, the patient group was dichotomized in two categories: with lymphedema and without lymphedema 1 month after completion of TAC (T3).

The Lymph-ICF questionnaire for the upper extremity was used to get insight in impairments in functions and limitations in activities in daily life [19]. The Lymph-ICF is a quality of life questionnaire developed for patients with lymphedema, with 29 items over five domains: physical function, mental function, household activities, mobility activities, and social activities. Each item can be scored between 0 and 100 on a horizontal line of 100 mm. Domain scores and the total score can be calculated from the items, both resulting in a score between 0 and 100. A higher score means more impairment in functions or limitation in activities: scores under 25 indicate a minor problem, scores between 25 and 50 a moderate problem, and scores more than 50 a severe problem. Measurement properties of the Lymph-ICF have been studied before and showed a fair to excellent reliability for all scales (*r* = 0.65–0.93) compared to volume measurements [19]. Patients filled in the Lymph-ICF questionnaire at every measurement point. To get insight in recovery after TAC, 3 months after the last TAC (T4), the Lymph-ICF questionnaire was sent to the patient for a final measurement.

### Statistical analysis

Descriptive analyses were used to describe patient characteristics, treatment characteristics, the number of patients with lymphedema, and the scores of the Lymph-ICF questionnaire in total and its domains.

To analyze differences in volume and consequences in daily life over time (total score and domain scores on the Lymph-ICF questionnaire) and to identify treatment and patient characteristics as predictors for changes in volume, linear mixed-effect models were used. We estimated a random intercept model with volume difference as dependent variable, and we estimated separate models with total score and domain scores of the Lymph-ICF questionnaire as dependent variables. To indicate predictors for lymphedema, univariate analysis was used to analyze the association between volume difference and type of surgery, surgery on dominant side, axillary lymph node dissection, tumor stage, nodal stage (N0 versus N1, N2, and N3), tumor grade (T1 versus T2 or 3), adjuvant radiotherapy, radiotherapy to the axilla or supraclavicular region, and change of BMI between T0 and T3. Variables with an association *p* < 0.20 in the univariate analysis were included into the model as independent variables.

To analyze the association between volume differences and impairments in functions and limitations in activities after completion of TAC, Pearson’s correlation coefficients between volume and the outcomes of the Lymph-ICF questionnaire (total score and domain scores) were calculated for the total population. To analyze the relation between scores of the Lymph-ICF questionnaire and lymphedema, patients were dichotomized in patients without lymphedema and patients with lymphedema at 1 month after completion of TAC (T3). In both groups, the association between the volume and the scores of the Lymph-ICF questionnaire, its five domains, and individual items of the physical function domain and mobility activities domain (T3 and T4) were analyzed, using Spearman’s correlations.

Correlations between measurement outcomes were interpreted as follows: *r* between 0.40 and 0.75 is fair to good; *r* > 0.75 is excellent [20].

For statistical analysis, SPSS version 22 was used.

## Results

**Fig. 1:**
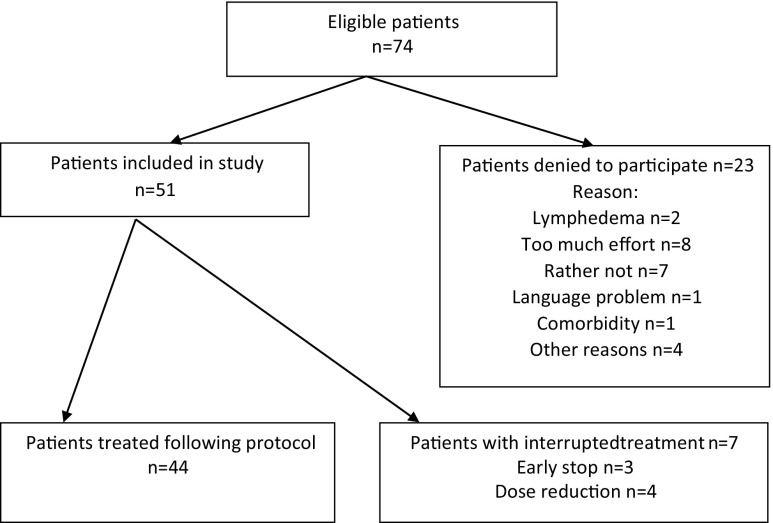
Flowchart patient inclusion for measuring before, during, and after adjuvant TAC

A total of 74 patients scheduled for adjuvant TAC were invited to participate in the study. Fifty-one patients consented to participate, one of these was male. Mean age of the included patients was 51.3 years (30–68; SD 8.5). Patient and tumor characteristics of these patients are summarized in Table 1. Two patients switched treatment to FEC (5-fluorouracil, epirubicin, and cyclophosphamide) after the third and fourth cycles. One patient was treated without docetaxel in the fourth cycle of TAC and stopped after this cycle. Finally, 48 patients completed six cycles of TAC and all follow-up measurements. Four of these 48 patients were treated with reduced dose (75%) after the third (*n* = 2 patients), fourth (*n* = 1 patient), or fifth cycle (*n* = 1 patient) (see Fig. 1).

**Table 1:** Baseline patient, tumor, and treatment characteristics

		Number of patients	Frequency (%)
Sexes	Female/male	50/1	98/2
Age	(Mean-SD)	51.3	8.5
BMI	(Mean-SD)	26.26	4.44
Surgery	Dominant arm	25 (3 left, 22 right)	49
	Non-dominant arm	26 (3 left, 23 right)	51
	Mastectomy/lumpectomy	27/24	52.9/47.1
	ALND/SNB	16/44	31.4/86.3
Tumor size	≤ 2 cm	22	43.1
	2–5 cm	26	50.9
	> 5 cm	3	5.9
Histology	Ductal carcinoma	46	90.2
	Lobular carcinoma	4	7.8
	Other	1	2
Tumor stage	Tis	1	2.0
	T1	3	5.9
	T1b	2	3.9
	T1c	18	35.3
	T1(m)	2	3.9
	T2	17	33.3
	T2(m)	7	13.7
	T3	1	2.0
Nodal stage	No	10	19.6
	N0(i+)-isolated tumor cells	11	21.6
	N1	10	19.6
	N1(mi) micrometastasis	11	21.6
	N(1a)	7	13.7
	N(2a)	2	3.9
Tumor grade	T0	1	2.0
	T1	4	7.8
	T2	27	49.0
	T3	19	39.2
Estrogen receptor	Positive/negative	45/6	85.2/14.8
Progesterone receptor	Positive/negative	34/17	66.7/33.3
Radiotherapy		33	64.7
	Boost tumor bed	18	35.3
	Axillary/supraclavicular radiation	11	21.6

*ALND* axillary lymph node dissection, *BMI* body mass index, *SNB* sentinel node biopsy, *cm* centimeter, *SD* standard deviation

### Changes in arm volume and lymphedema measured by tape measurement

Mean volume did not change during treatment but increased significantly from 2.3% at T0 to 5.1% at 1 month after completion of TAC (T3) (*p* = 0.01) (see Table 2). In total, 15 patients showed increased volume of ≥ 10% difference in at least one measurement point. In three of the six patients, swelling was deemed transient: volume decreased under the cutoff point of 10% volume difference within the study period, one of them without treatment. Fulfilling our definition, lymphedema was observed first in six patients during TAC treatment. These patients were referred for lymphedema treatment: two patients were indicated at baseline (T0), two before the second (T1), and two before the fourth cycle during chemotherapy (T2). One month after treatment (T3), lymphedema was observed in 12 out of 48 patients (25%).

**Table 2:** Lymphedema measurements with tape at 10 cm distance, calculated as relative volume difference between affected and unaffected upper extremity

	Mean (%)	Standard error	95% Confidence Interval	Significance
T0	2.282	.925	0.458–4.107	
T1	2.180	.925	0.356–4.005	0.938
T2	2.689	.944	0.827–4.550	0.759
T3	5.708	.924	3.872–7.633	0.010*

*T0* before TAC 1, *T1* before TAC 2, *T2* before TAC 4, *T3* 1 month after TAC 6

*Significance at *p* < 0.05

### Predictors for of lymphedema

Axillary lymph node dissection, nodal stage, axillary radiation, and difference in BMI identified between 1 month after completion of TAC (T3) and baseline (T0) were variables with a correlation (*p* < 0.10) with volume at T3 in the univariate analysis. The linear mixed-effect model showed that axillary lymph node dissection was the only factor significantly associated with increased volume (ES 2.9%; 95% CI 0.02–5.7; variance 9.5%, *p* < 0.05 (see Table 7); odds ratio 12.4; 95% CI 2.6–58.3; *p* < 0.01).

### Impairments in functions and limitations in activities in daily life

Mean total score of the Lymph-ICF questionnaire showed an increase from 14.6 at baseline (T0) to 19.5 at 1 month after completion of TAC (T3) and subsequently decreased to 16.5 at 3 months after completion of TAC (T4). Longitudinal analysis showed no significant changes over time except in the domain physical functioning. One month after TAC completion, physical functioning showed a significant increase (*p* = 0.01) compared to T0–T2. Three months after, TAC physical functioning improved, but scores remained higher compared to T0 (*p* < 0.05) (Table 3).

**Table 3:** Lymph-ICF questionnaire and its domains at baseline before chemotherapy (T0), during chemotherapy before TAC 2 (T1) and before TAC 4 (T2), 1 month after completion of TAC 6 (T3), and 3 months after completion of TAC 6 (T4)

Measurement point	Number	Mean	Standard Error	Significance	95% confidence interval
Total score					
T0	51	14.63	2.20		10.29–18.97
T1	51	11.31	2.20	0.29	6.97–15.66
T2	49	12.98	2.25	0.60	8.55–17.41
T3	48	19.46	2.27	0.13	14.98–23.93
T4	48	16.52	2.27	0.55	12.05–21.00
Physical function					
T0	51	9.80	2.19		5.49–14.12
T1	51	6.90	2.19	0.35	2.58–11.22
T2	49	9.96	2.24	0.96	5.55–14.37
T3	48	17.79	2.26	0.01*	13.34–22.24
T4	48	16.67	2.26	0.03*	12.22–21.12
Mental function					
T0	51	11.75	2.23		7.36–16.13
T1	50	7.74	2.25	0.21	3.31–12.17
T2	49	8.18	2.27	0.26	3.71–12.66
T3	48	11.90	2.94	0.96	7.38–16.42
T4	48	9.46	2.94	0.48	4.94–13.98
Household activities					
T0	51	14.98	2.87		9.79–20.63
T1	51	13.71	2.87	0.75	8.06–19.35
T2	49	12.02	2.93	0.47	6.26–17.78
T3	48	21.38	2.96	0.12	15.55–27.20
T4	48	16.81	2.96	0.66	10.99–22.63
Mobility activities					
T0	51	18.14	2.74		12.73–23.54
T1	51	13.73	2.74		8.32–19.13
T2	49	14.00	2.80	0.26	8.49–19.52
T3	48	20.58	2.83	0.29	15.01–26.12
T4	48	19.31	2.83	0.54	13.74–24.89
Social activities					
T0	51	19.20	3.19		12.92–25.47
T1	51	16.45	3.19	0.54	10.18–22.73
T2	49	19.35	3.25	0.97	12.94–25.75
T3	48	23.79	3.28	0.32	17.32–30.26
T4	48	18.21	3.28	0.83	11.74–25.68

*Significance at *p* < 0.05

The number of patients with moderate to severe problems (scores ≥ 25) on the Lymph-ICF questionnaire decreased between T3 and T4. Looking at the number of patients without lymphedema and with lymphedema, relatively more patients with lymphedema experienced problems, especially in total score, physical functioning, mobility activities, and social activities, 1 and 3 months after treatment (T3, T4). Patients without lymphedema reported more problems with mental functions 1 month after treatment (see Table 4). One month after completion of TAC, in patients with lymphedema, a significant association was found between volume and the Lymph-ICF total score (*r* = 0.66), the physical function domain (*r* = 0.77), the item scores for heaviness (*r* = 0.83) and swelling (*r* = 0.71), and the mobility activities domain (*r* = 0.66), as well the items activities above the head (*r* = 0.71) and cycling (*r* = 0.72). Three months after completion of TAC, in patients with lymphedema, the significant correlation between relative volume at T3 and the total Lymph-ICF score (*r* = 0.70) and mobility activities (*r* = 0.62) remained (see Table 5).

**Table 4:** Patients with moderate (≥ 25; < 50) and severe (≥ 50) problems indicated by the Lymph-ICF questionnaire after treatment with TAC

	One month after completion (T3)			Three months after completion (T4)		
Reported score	≥ 25; < 50	≥ 50	total	≥ 25; < 50	≥ 50	total
Total score	*12*	*5*	*17*	*6*	*4*	*10*
Without lymphedema	9	3	12	4	3	7
With lymphedema	3	2	5	2	1	3
Physical function	*10*	*4*	*14*	*6*	*4*	*10*
Without lymphedema	5	3	8	4	3	7
With lymphedema	5	1	6	2	1	3
Mental function	*3*	*3*	*6*	*–*	*4*	*4*
Without lymphedema	2	3	5	–	3	3
With lymphedema	1	–	1	–	1	1
Household activities	*11*	*6*	*17*	*6*	*5*	*11*
Without lymphedema	9	4	13	5	3	8
With lymphedema	2	2	4	1	2	3
Mobility activities	*9*	*7*	*16*	*11*	*6*	*17*
Without lymphedema	6	5	11	9	4	13
With lymphedema	3	2	5	2	2	4
Social activities	*10*	*8*	*18*	*7*	*8*	*15*
Without lymphedema	7	5	12	7	5	12
With lymphedema	3	3	6	–	3	3

Patients without lymphedema *n* = 36; patients with lymphedema *n* = 12

Scores in italic: total number of patients with problems on the mentioned item

**Table 5:** Correlations between volume measured with tape measurement and Lymph-ICF questionnaire 1 month after completion of TAC (T3) in patients without and with lymphedema

	Relative volume	Total score	Physical function	Mental function	Household activities	Mobility activities	Social activities
Patients without lymphedema (*n* = 36)							
Relative volume	1.000						
Total score	− 0.341*	1.000					
Physical function	− 0.014	0.696**	1.000				
Mental function	− 0.368*	0.660**	0.324	1.000			
Household activities	− 0.479**	0.885**	0.526**	0.617**	1.000		
Mobility activities	− 0.380*	0.853**	0.665**	0.496**	0.833**	1.000	
Social activities	− 0.305	0.828**	0.326	0.657**	0.752**	0.689**	1.000
Patients with lymphedema (*n* = 12)							
Relative volume	1.000						
Total score	0.658*	1.000					
Physical function	0.768**	0.676*	1.000				
Mental function	0.445	0.667*	0.320	1.000			
Household activities	0.449	0.844**	0.488	0.557	1.000		
Mobility activities	0.660*	0.865**	0.602*	0.585*	0.849**	1.000	
Social activities	0.180	0.739**	0.145	0.367	0.684*	0.607*	1.000

*r* between 0.40 and 0.75 is fair to good; *r* > 0.75 is excellent

*Significance < 0.05 (two-tailed); **significance < 0.01 (two-tailed)

## Discussion

During cytotoxic treatment with TAC, we observed no significant changes in volume between upper extremities in the total study population. However, 1 month after completion of TAC, volume was increased significantly, and 25% of the patients had developed a volume difference over 10%, defined as lymphedema. Also, in this population, axillary lymph node dissection was predictive for development of lymphedema, as was reported in earlier studies as well [6, 21]. The Lymph-ICF questionnaire showed significant impairments in the physical function domain at 1 and 3 months after completion. One month after treatment, 17 patients showed at least moderate problems on the total score of the questionnaire and reported problems in physical function, household activities, mobility activities, and social activities. We observed a small decline in the number of patients with health problems between 1 and 3 months after completion of TAC.

Reported prevalence of lymphedema, measured at a comparable time point after surgery in recent studies on breast cancer, was comparable with our study at baseline [13, 14, 22, 23]. DiSipio et al. described in their systematic review a prevalence of lymphedema of 10.3% (95% CI 6.2–16.7) at the same time point as T3 in our study, after completion of cytotoxic treatment [1]. In relation to their study, the prevalence of lymphedema at the endpoint in our study is higher. This could be the effect of the adjuvant treatment with TAC as suggested in a recent study indicating docetaxel as important risk factor for onset of lymphedema, with a chance of developing lymphedema being 4.8 times higher when compared to other treatment regimens [10] and reported in earlier studies as well [7–9, 11–14]. Compared to our study, earlier studies on TAC as a risk factor for lymphedema reported a higher prevalence of lymphedema over two or more years after treatment with TAC with 33.5% [12], 42.2% [14], and 32% after treatment, declining to 23% at 6 months [13]. Although bio-impedance spectroscopy (BIS) measures extracellular fluid more adequately and good correlations between volume measurements and BIS were found in case of swelling [18, 24–26], we decided not to add BIS in our measurement protocol to decrease patient load during the study. As results from different studies can be compared, we decided to decrease patient load during the study. The somewhat lower prevalence of lymphedema (25%) in our study may be the result of lymphedema treatment of patients with a volume difference over 10%, later onset of lymphedema, as well as recent developments in supportive care encouraging patients to stay active during treatment with at least 30 min of moderate daily physical activity [27]. Referral to physical therapy or lymphedema treatment was reported in one other study [10]. Our analysis of predictive factors for development of lymphedema early after treatment with TAC confirms the findings of Lee et al.: axillary lymph node dissection is an important risk factor for development of lymphedema [13].

Concerning the Lymph-ICF questionnaire, problems were apparent over a longer period, in patients with and without lymphedema. Looking to the results in Table 4, it can be observed that the number of patients with problems on the Lymph-ICF questionnaire differs between groups, while Tables 5 and 6 point out that volume increase is associated with more problems at the Lymph-ICF questionnaire. This means that all patients, with or without lymphedema, experienced problems and the Lymph-ICF questionnaire revealed health problems in the whole study-population. However, lymphedema increased the scores. Compared to the literature, in a study with FEC (fluorouracil, epirubicin, and cyclophosphamide), one third of the patients still had problems as well [28] and problems related to work were reported in a recent systematic review [4]. Especially mobility activities are an indication for decreased social contacts and participation in community life and work, which are important factors for quality of life [29, 30]. Moderate- to high-intensity exercises during chemotherapy could have limited the decrease in activities during adjuvant treatment, as was reported in an earlier study in a comparable population, reporting significant positive effects of exercise on physical function, fatigue, and chemotherapy completion rates [31].

**Table 6:** Correlations between relative volume measured with tape measurement and items of the Lymph-ICF questionnaire 1 month after completion of TAC (T3) in patients with lymphedema (*n* = 12)

	Relative volume	Heaviness	Swelling	Strength	Tense feeling	Activities above head	Lifting heavy objects	Sleeping on affected side	Working on computer	Walking > 2 km	Cycling
Relative volume	1.000										
Heaviness	0.830**	1.000									
Stiffness	0.516	0.525									
Swelling	0.709**	0.872**	1.000								
Strength	0.550	0.797**	0.753**	1.000							
Tense feeling	0.421	0.616*	0.796**	0.753**	1.000						
Activities above head	0.705*	0.774**	0.814**	0.778**	0.618*	1.000					
Lifting heavy objects	0.564	0.613*	0.434	0.648*	0.219	0.734**	1.000				
Sleeping on affected side	0.348	0.287	0.113	0.644	− 0.157	0.673*	0.850**	1.000			
Working on computer	0.500	− 1.000**	− 0.500	0.500	0.500	0.500	0.500	0.500	1.000		
Waling > 2 km	0.295	0.116	0.095	0.363	0.329	0.286	0.277	0.540	1.000**	1.000	
Cycling	0.723*	0.492	0.322	0.610	0.340	0.729*	0.732*	0.962**	1.000**	0.948**	1.000

*r* between 0.40 and 0.75 is fair to good; *r* > 0.75 is excellent

*Significance < 0.05 (two-tailed); **significance < 0.01 (two-tailed)

The item scores of heaviness and swelling were significantly associated with volume in patients with lymphedema. These self-reported outcomes can indicate lymphedema of the affected upper extremity [26, 32, 33], can be used as a patient’s reported outcome for lymphedema, and are supportive in the clinical decision making on volume measurement and referral for lymphedema treatment. When moderate to severe problems are reported on the Lymph-ICF questionnaire, referral to specialized health care should be considered to improve functions and activities as soon as possible [34, 35]. In agreement with a previous systematic review of the literature [6], many factors play a role in complaints of patients after medical treatment for breast cancer. For the patient as well as the healthcare provider, it is important to know the origin of the complaints. It is unclear why the item cycling within the domain mobility activities has high scores in most of the patients; probably, this can be related to reduced cardiovascular function as an adverse effect of TAC [16] and to fibroses of the breast in patients treated with lumpectomy and radiotherapy (OR 7) [36] or pain after radiotherapy of the chest wall, as was reported by Levangie et al. in 26% of the patients, leading to reduced daily activities [5]. Limitations in activities above the head can be related to declined shoulder mobility, which is often described as adverse effect of axillary node dissection and/or axillary radiotherapy, or declined muscle strength or shoulder coordination, described as adverse effects of axillary node dissection and chemotherapy [6, 37].

This is the first study describing arm volume during TAC as an objective measurement of lymphedema in combination with patient reported outcome measures on physical and mental function, household activities, mobility activities, and social activities. However, some limitations should be considered.

Preoperative measurements were not incorporated in our measurement protocol, although such measurements have been recommended in the literature [38–42]. However, the first measurement was started within 3 months post-operatively and lymphedema is rarely reported within the first months post-operatively. Moreover, this time point was found as significant predictor by Sun et al. for over- and underdiagnoses [40]. Lymphedema defined as relative volume change (RVC) compared to baseline as used by Sun et al. is a different definition compared to our definition using relative volume difference (RVD) between extremities. We used RVD following the commonly used definition in the literature [1, 6]. Future studies need to point out which of the definitions is most adequate. Furthermore, patient and treatment characteristics should be analyzed in relation to volume change during a longer follow-up with more measurement occasions. A rather conservative cutoff point of 10% between both upper extremities was defined as lymphedema, based on the smallest detectable change in tape measurement (6.6%, with excellent interrater reliability (ICC inter 0.98) [18]. Probably, a cutoff point of 5% would have increased the number of patients with (subclinical) lymphedema and transient edema.

Although the sample was large enough to distinguish changes in volume difference during the study period, a larger sample size and a longer follow-up might have indicated more risk factors for development of lymphedema over time, and a higher prevalence of lymphedema, as reported in earlier studies on TAC and docetaxel [8–12, 14, 40]. As no measurements were performed between cycles 4 and 6, the exact time point of onset of lymphedema cannot be determined. A longer follow-up would have enabled the distinction between transient swelling from persistent lymphedema. Swelling can be transient as a result of spontaneous recovery or by intervention, as reported earlier [1, 8, 10, 13, 14]. In future research and in clinical practice, volume measurements should be taken at baseline and at least in the first follow-up visit after completion of TAC. Follow-up should be extended to differentiate between transient swelling and lymphedema, reporting lymphedema treatment as well.

Altogether, monitoring swelling seems to have added value and seems to be clinically relevant.

## Conclusion

In our population, arm volume increased significantly 1 month after treatment with TAC and in 12 out of 48 patients (25%) relative volume difference increased over 10%. Axillary lymph node dissection was predictive for development of lymphedema. After treatment with TAC, all patients, but more patients with lymphedema, perceived difficulties in activities in daily life.

## Appendix

**Table 7:** Fixed effects of the linear mixed-effect model in relation to predictive factors for increase of relative volume at T3

Parameter	Estimate	Standard error	*p* value	95% confidence interval
Intercept	1.68	0.63	0.01	0.44–2.93
ALND	2.86	1.17	0.05*	0.02–5.69
Nodal stage	1.44	1.09	0.20	− 0.72–3.60
Axillary radiation	0.65	2.75	0.81	− 4.78–6.08
BMI T3-T0	− 0.00	0.00	0.45	− 0.01–0.00
ALND * nodal stage	− 0.26	3.16	0.94	− 8.82–8.30
ALND * axillary radiation	297.67	181.87	0.10	− 61.30–656.64
ALND * BMI T3-T0	2.02	0.74	0.07	− 0.26–4.30
Nodal stage * axillary radiation	− 4.14	3.28	0.21	− 10.62–2.34
Nodal stage * BMI T3-T0	0.00	0.48	1.00	− 0.95–0.96
Axillary radiation * BMI T3-T0	0.00	0.00	0.89	− 0.01–0.01
ALND * nodal stage * axillary radiation	− 298.79	187.54	0.11	− 668.96–71.37
ALND * nodal stage * BMI T3-T0	− 0.80	1.87	0.69	− 6.04–4.45
ALND * axillary radiation * BMI T3-T0	310.77	189.37	0.10	− 62.99–684.54
Nodal stage * axillary radiation * BMI T3-T0	− 0.00	0.48	1.00	− 0.954–0.95

*ALND* axillary lymph node dissection, *BMI* body mass index
